# The invasion of *Euphorbia jolkinii* is mediated through the regulation of nitrogen transformation by functional microbial abundance in rhizosphere soils

**DOI:** 10.3389/fmicb.2026.1757844

**Published:** 2026-02-24

**Authors:** Xue Xiao, Qiongmei Niu, Kai Zhou, Laixiang Ma, Zhili Zhao, Jiangui Zhang, Xiaohui Chu, Guilian Shan

**Affiliations:** Faculty of Animal Science and Technology, Yunnan Agricultural University, Kunming, China

**Keywords:** biological invasion, *Euphorbia jolkinii*, N transformation, N-acquiring microbiome, nitrogen cycle

## Abstract

**Introduction:**

*Euphorbia jolkinii* Boiss. is a native invasive weed. Its invasion altered microbial composition, total nitrogen (TN) and available nitrogen (AN). However, the mechanisms influencing N transformation remain unclear. Particularly, the roles of the microbiome and genes in mediating N transformations to facilitate *E. jolkinii* invasion remain poorly understood. Therefore, the primary objectives of this study were to evaluate how *E. jolkinii* invasion affects N transformation, microbial interactions, and key genes associated with AN accumulation.

**Methods:**

We compared three patches (non-invaded, lightly, and heavily invaded patches of *E. jolkinii*) by analyzing rhizosphere soils of *E. jolkinii* and *Poa crymophila* Keng. Integrating soil physicochemical indices with metagenomic sequencing, we investigated the relationships among microbial communities, gene abundance, and N transformation.

**Results:**

With *E. jolkinii* increasing invasion intensity, N accumulation and transformation rates were significantly reduced in the rhizosphere of *P. crymophila* but enhanced in that of *E. jolkinii*, particularly for AN. Metagenomic analysis revealed that the invasion and expansion of *E. jolkinii* promoted functional adaptation of the microbial community, particularly by enriching the N cycling-related genes and increasing their relative abundance in the rhizosphere soil of *E. jolkinii*. Moreover, it inhibited the accumulation of N transformation functional genes in the rhizosphere soil of the companion plant, *P. crymophila*. Structural equation modeling identified Nitrospirota, *Edaphobacter*, *Anaeromyxobacter*, and soil N transformation rates as key drivers of AN accumulation.

**Discussion:**

*E. jolkinii* facilitated N accumulation in its rhizosphere by modulating N-transforming microbes and key functional genes, underscoring one of its invasive advantages.

## 1 Introduction

Northwestern Yunnan Province lies on the southern fringe of the Tibetan Plateau, an area characterized by vast, contiguous stretches of natural subalpine meadows. These meadows not only provide a crucial material foundation for the survival and development of the local Tibetan communities but also function as a vital ecological barrier, helping to safeguard the environmental security of the Tibetan Plateau region. However, due to irrational utilization and environmental changes, the subalpine meadows of northwestern Yunnan have exhibited significant degradation characteristics, including the invasive expansion of toxic weeds and a sharp decline in the quantity of edible forage. The toxic weeds are primarily *Euphorbia jolkinii*, *Ligularia dictyoneura*, *Ligularia vellerea*, and *Stellera chamaejasme* (Zhao et al., 2021), among which *E. jolkinii* has the widest distribution and the highest abundance. Previous results indicate that *E. jolkinii* is now widespread across degraded alpine meadows, with an average density of 2.2 clumps/m^2^ and a maximum density of 11.4 clumps/m^2^. In areas where *E. jolkinii* populations are densely concentrated, the number of plant species in the grassland sharply declines. The community is dominated only by *E. jolkinii*, *Potentilla fulgens*, *Spiranthes sinensis*, *L. dictyoneura*, *P. crymophila*, *Arundinella hooker*, *Carex tristachya*, and *Artemisia stechmannian* (Yang et al., 2025). Moreover, the yield of high-quality forage grasses such as *P. crymophila*, *A. hookeri*, and *C. tristachya* significantly decreases, while the density and biomass of *E. jolkinii* markedly increase. Consequently, *E. jolkinii* has become the dominant species in the community. *E. jolkinii* is highly toxic, and its accidental ingestion by livestock can cause severe poisoning. Therefore, the spread and invasion of this plant pose a serious threat to the development of local grassland livestock husbandry and the conservation of biodiversity. To mitigate *E. jolkinii* invasion effectively, it is essential to elucidate its underlying invasion mechanisms, particularly its competitive dominance over native vegetation in soil nutrient acquisition.

*Euphorbia jolkinii* Boiss. is a toxic perennial herbaceous plant belonging to the Euphorbiaceae family. It is widely distributed in the subalpine meadows, slopes, and shrublands at altitudes of 2,380–3,300 m in Shangri-La, Northwestern Yunnan (Xiao et al., 2025). Compared with other species in the community, *E. jolkinii* exhibits strong resistance to cold and drought, high reproductive and spatial expansion capacity, giving it a competitive advantage in acquiring soil moisture and nutrients (Li et al., 2021; Zhang et al., 2020). These traits enable it to transform invaded grasslands into degraded grasslands dominated by *E. jolkinii*. Its spread and expansion are important indicators of degradation or successional change in subalpine meadows, seriously impacting the local grassland ecosystem (Chen et al., 2024). However, preliminary studies have found that *E. jolkinii*-dominated grasslands do not exhibit common degradation characteristics such as soil exposure, water and soil erosion, or soil deterioration. On the contrary, *E. jolkinii* invasion significantly increases the total biomass and surface cover, promotes the growth and reproduction of soil bacteria, fungi, and actinomycetes, and enhances soil microbial biomass carbon (MBC) and microbial biomass nitrogen (MBN) (Yang et al., 2025). It also markedly increases the contents of available NH_4_^+^–N and NO_3_^−^–N (Yang et al., 2024). These alterations in soil N transformation and microbial composition may play a pivotal role in determining the success of its invasion.

Recent research has focused on the dynamic interactions between invasive species and soil microbial communities (Zhao M. X. et al., 2024), demonstrating that invasive plants can facilitate their successful invasion by altering these communities (Zhang et al., 2023). For example, invasive plants can significantly influence soil nutrient availability and create a more conducive soil habitat by modifying the microbial community composition (Zhao et al., 2019). Soil microbial communities serve as integral connectors within key ecological functions, including nitrogen-cycling processes (Chaudhary et al., 2023). Nitrogen (N) is a limiting nutrient for plant growth in various ecosystems (Jin et al., 2024). The N cycle comprises several critical processes, including the plant N uptake and utilization, N input through litterfall deposition, soil microbial mediated N fixation, and N release (Jin et al., 2024). The soil N cycle is intricately linked to N-fixing, ammonifying, nitrifying, and denitrifying bacteria (Yu et al., 2020), as several critical processes in this cycle are primarily mediated by these organisms. For instance, N fixation from the atmosphere, denitrification, and ammonium oxidation are key transformations predominantly driven by microbial activity (Hartmann and Johan, 2022). The composition and abundance of these microbial communities are substantially influenced by plant invasion, thereby influencing N-cycling processes (Edwards et al., 2022; Ren et al., 2024). For example, cheatgrass invasion enhances N fixation and reduces N loss by promoting colonization of N-fixing bacteria in the rhizosphere while simultaneously suppressing denitrifier activity, ultimately leading to elevated soil total N (TN) concentrations (Reitstetter et al., 2022). *Mikania micrantha* invasion significantly influences the N cycle and accumulation of available N (AN) by modifying both the abundance and community structure of ammonia-oxidizing archaea (Yu et al., 2020). The supply of AN in the rhizosphere soil primarily depends on microbial communities that transform inert N compounds into accessible forms. Therefore, microbially mediated N transformations are pivotal determinants of N utilization by invasive plants (Gabriela and McCary, 2024).

The invasion and expansion of *E. jolkinii* in the alpine meadows altered grassland vegetation, soil environment, and microbial communities. However, how does its invasion influence soil N transformation processes, as well as the abundance and activity of key soil microorganisms? Furthermore, what are the specific roles of the key soil microbes in mediating N transformations? We hypothesize that *E. jolkinii* enhances soil N availability by enriching for microbial communities involved in the N cycle, leading to distinct microbial composition and higher N-transformation rates relative to the native *P. crymophila* rhizosphere. Given that *E. jolkinii* is a non-leguminous species, we further hypothesize that the increased N availability in its rhizosphere soil is primarily driven by nitrification rather than N fixation.

To investigate these scientific questions, we selected the Tuo Munan Group National Grassland Fixed Monitoring Site in Shangri-La City, Northwestern Yunnan as the research plot. This site is a subalpine meadow dominated by the native grass *P. crymophila*, which is invaded by *E. jolkinii*. Based on the patchy distribution of *E. jolkinii* in the monitoring site, sampling units were designated as non-invasion, lightly, and heavily invaded patches of *E. jolkinii*. The study aimed to investigate how soil N transformation and key functional microbial communities respond to *E. jolkinii* invasion, and to analyze the underlying mechanisms, with a focus on microbial feedback regulation of the soil N cycle. The findings may provide guidance for formulating adaptive management strategies for grasslands invasion by *E. jolkinii* in northwestern Yunnan.

## 2 Materials and methods

### 2.1 Experimental plot and research subjects

Based on previous research, a national-level fixed grassland monitoring site in Tuomunan, Shangri-La City, northwestern Yunnan, was selected as the experimental plot. The plot is situated at latitude N27°29′0″ and longitude E99°52′16″, with an elevation of 3,240 m. The climate is classified as a cold-temperate mountainous monsoon climate, characterized by relatively low temperatures, with an average annual temperature of 5.8 °C. Currently, the vegetation in the plot is growing well and has 100% vegetation cover. The original dominant species of the meadow was *P. crymophila*. Currently, the invasive species *E. jolkinii*, which exhibits a patchy distribution, has become dominant across the plot.

Based on the patchy distribution of *E. jolkinii* in the monitoring plot, three patch types were designated as sampling units: non-invaded patches (0% coverage of *E. jolkinii*, N), lightly invaded patches (coverage of *E. jolkinii* <10%, L), and heavily invaded patches (coverage of *E. jolkinii* >40%, H). To ensure spatial independence, a minimum interpatch distance of 50 meters was maintained between sampling units. Each patch type was replicated three times, with each replicate covering an area exceeding 30 m^2^. The basic characteristics of the different patch types (sampling units) are summarized in Supplementary Table S1.

The rhizosphere soils of *E. jolkinii* and *P. crymophila* were selected as the research subjects in this study. *E. jolkinii*, a toxic weed of the Euphorbiaceae family, is widely distributed in the subalpine meadows of northwest Yunnan and is recognized as an invasive species in grassland ecosystems. *P. crymophila*, a dominant companion plant in the invasive patches of *E. jolkinii*, is recognized as a native plant in grassland ecosystems. In the alpine meadows of Shangri-La, *E. jolkinii* and *P. crymophila* coexist in the same habitat and share similar ecological niches. Given the limiting similarity hypothesis, which posits that species with overlapping ecological niches are likely to engage in intense resource competition, we hypothesized that these two plant species compete for shared environmental resources. Therefore, we selected these two species as the primary focus of our study.

### 2.2 Soil sample collection

**Figure 1:**
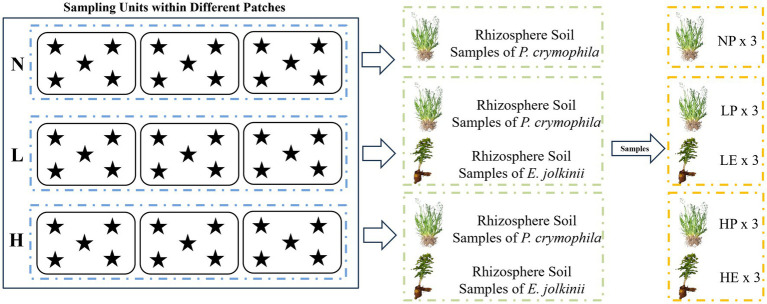
Schematic diagram of rhizosphere soil sampling units and sampling points. N, *E. jolkinii* non-invasion patches; L, *E. jolkinii* lightly invaded patches; H, *E. jolkinii* heavily invaded patches.

**Figure 2:**
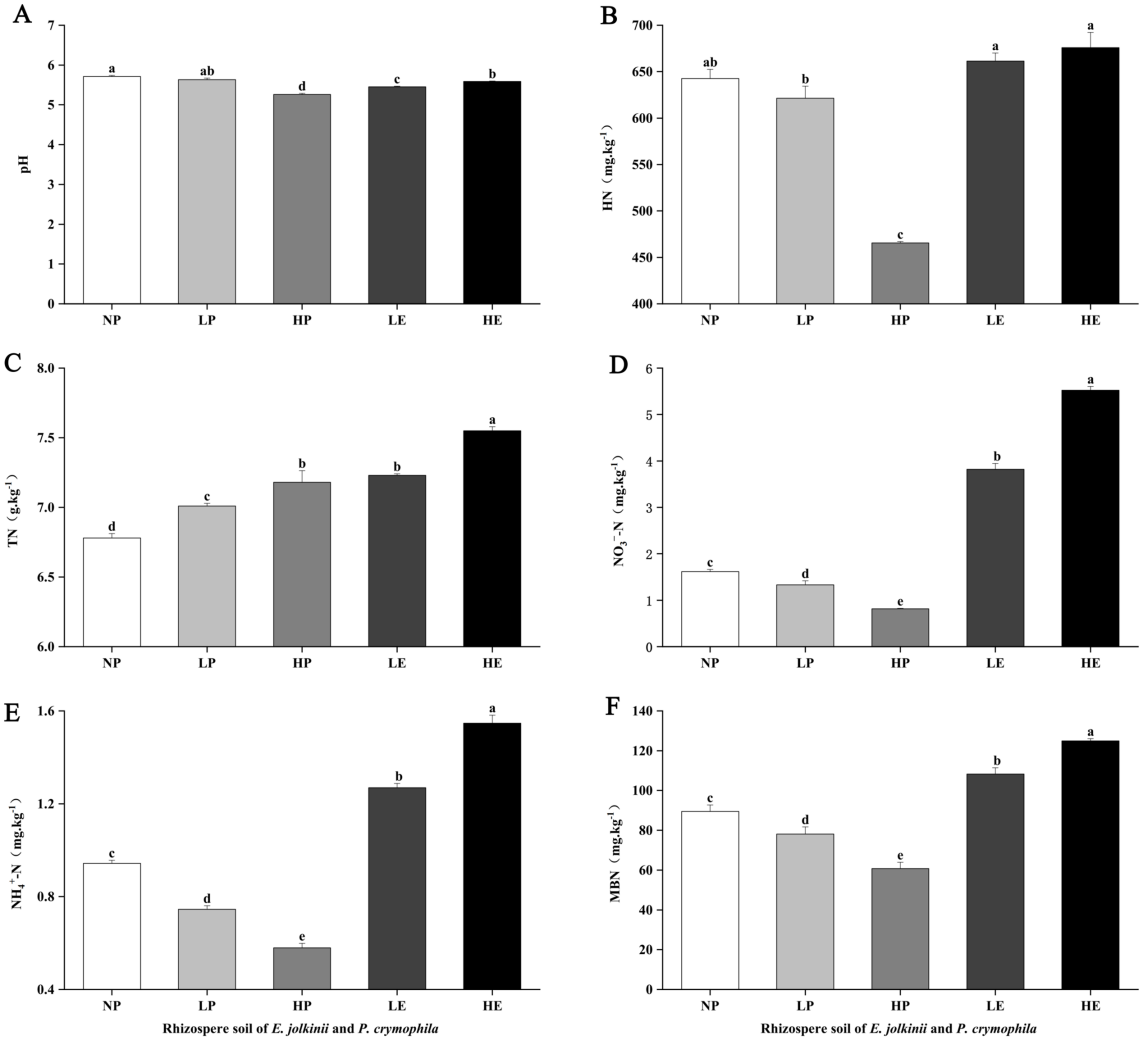
The impact of *E. jolkinii* invasion on pH and N components. Changes in pH **(A)**; hydrolyzable nitrogen (HN) **(B)**; total nitrogen (TN) **(C)**; NO_3_^−^–N **(D)**; NH_4_^+^–N **(E)**; and microbial biomass nitrogen (MBN) **(F)** of *E. jolkinii* and *P. crymophila* rhizosphere soils (mean ± standard error (SE), *n* = 3). Different letters above bar plots indicate significant differences (Duncan test, *p* < 0.05). NP, *P. crymophila* rhizosphere soils of *E. jolkinii* non-invasion patches; LP, *P. crymophila* rhizosphere soils of *E. jolkinii* lightly invaded patches; LE, *E. jolkinii* rhizosphere soils of *E. jolkinii* lightly invaded patches; HP, *P. crymophila* rhizosphere soils of *E. jolkinii* heavily invaded patches; HE, *E. jolkinii* rhizosphere soils of *E. jolkinii* heavily invaded patches.

As illustrated in Figures 1, 2, three sampling units were randomly selected within each plot as replicates. Within each sampling unit, five sampling points were chosen for soil collection. Soil from these five points of each plant species was combined to form one composite sample per unit. The rhizosphere soil was collected using the “root shaking method” (Bai et al., 2025). Briefly, 30–40 individual plants of *E. jolkinii* and *P. crymophila* were randomly selected at each sampling point. After gently shaking the roots to remove loosely adhering soil, rhizosphere soil was collected using a sterile brush. For each plant species, soil from the five points within a sampling unit was mixed to create a composite sample. Thus, each sampling unit yielded one composite replicate per species. All fresh rhizosphere soil was then sieved (2 mm mesh) to remove root debris. Processed samples were divided for different analyses: one portion was stored at 4 °C for subsequent assays of N transformation rates; another was immediately frozen at −80 °C for metagenomic sequencing; and the remainder was air-dried for the analysis of soil physicochemical properties and N fractions.

### 2.3 Soil environmental factors and N components analysis

Soil environmental factors were analyzed following the methods described by Zhang et al. (2020). Briefly, the pH was measured using a pH meter (Mettler-Toledo GmbH, Greifensee, Switzerland) with a 1:5 (w/v) soil-to-water suspension. Total carbon (TC) and organic carbon (OC) were determined following Chinese standard methods DZ/T 0279.25-2016 and DZ/T 0279.27-2016, respectively. Total phosphorus (TP) and available phosphorus (AP) were analyzed according to Chinese agricultural standards NY/T 88-1988 and NY/T 1121.7-2014, respectively. Similarly, total potassium (TK) and available potassium (AK) were determined following standards NY/T 87-1988 and NY/T 889-2004, respectively.

TN was determined using the Kjeldahl method, while hydrolyzable N (HN) was measured following the standard methods LY/T 1228-2015. The contents of NH_4_^+^–N and NO_3_^−^–N were determined using commercial kits, following the manufacturer’s protocols (Suzhou Gris Biotechnology Co., LTD, China). The microbial biomass nitrogen (MBN) was measured using the chloroform fumigation extraction method. Additional methodological details were provided in the Supplementary Tables S1, S2.

### 2.4 N transformation rates analysis

Transformation rates were determined according to the method described by Yuan C. Z. et al. (2024). For each replicate, a 50 g soil sample was weighed, transferred into an incubation bottle, and incubated in a constant-temperature incubator (GXZ-500B; Jiangnan Instrument Factory, Ningbo, China) for 28 d. To maintain consistent moisture levels throughout the incubation period, the bottles were reweighed every 3 d and distilled water was added as needed. The contents of TN, NH_4_^+^–N, and NO_3_^−^–N were measured at the beginning (day 0) and end (day 28) of the incubation. The organic nitrogen conversion rate (ONCR), net ammonification rate (AR), net nitrification rate (NR), and net mineralization rate (MR) were calculated as follows (Zhao Y. P. et al., 2024):


$$\text{ONCR}=[c{\left(\mathrm{TN}\right)}_{t1}-c{\left({\mathrm{NO}}_{3}^{-}\right)}_{t1}]-[c{\left(\mathrm{TN}\right)}_{t0}-c{\left({\mathrm{NO}}_{3}^{-}\right)}_{t0}]/(t1-t0)$$

$$\mathrm{AR}=[c{\left({\mathrm{NH}}_{4}^{+}\right)}_{t1}-c{\left({\mathrm{NH}}_{4}^{+}\right)}_{t1}]/(t1-t0)$$

$$\mathrm{NR}=[c{\left({\mathrm{NO}}_{3}^{-}\right)}_{t1}-c{\left({\mathrm{NO}}_{3}^{-}\right)}_{t1}]/(t1-t0)$$

MR=AR+NR

Where t0 and t1 represent the initial and final times, respectively, with c(TN)_t0_ and c(TN)_t1_ denoting the respective contents of TN at these time points. Similarly, c(NH_4_^+^)_t0_ and c(NH_4_^+^)_t1_ correspond to the NH_4_^+^–N contents measured at t0 and t1, with c(NO_3_^−^)_t0_ and c(NO_3_^−^)_t1_ indicating the content of NO_3_^−^–N at these two time points.

### 2.5 Metagenomic sequencing analysis

Soil DNA extraction and quality assessment procedures were conducted following the methods described by Zhu et al. (2024). Soil DNA was extracted from 0.5 g fresh soil using the HiPure Soil DNA Kit for Soil (Magen, Guangzhou, China). The DNA quality was assessed using Qubit (Thermo Fisher Scientific, Waltham, MA) and Nanodrop (Thermo Fisher Scientific, Waltham, MA).

Metagenome sequencing was performed on an Illumina NovaSeq X Plus PE150 platform (Illumina Inc., San Diego, CA, USA). All procedures for metagenomic sequencing, assembly of clean reads, and functional gene annotation were conducted by Gene Denovo Biotechnology Co., Ltd. Quality control of the metagenomic sequences was performed with Fastp ver. 0.18.0 to remove the adapters, reads with ≥10% unidentified nucleotides (N), low-quality reads with a length <50 bp, reads with an average quality score <20 and containing N bases (Zhu et al., 2024). The obtained clean reads were assembled to *de novo* assembly into contigs using Megahit ver.1.1.2 with optimized k-mer parameters. Functional annotation was performed using multiple complementary approaches. Sequences were aligned against the NCBI non-redundant protein (Nr) database, Kyoto Encyclopedia of Genes and Genomes (KEGG), and eggNOG databases using DIAMOND (v0.9.24) (Tang et al., 2024).

N-cycling functional genes were identified through protein localization using the NCyc database (Wang et al., 2023). These genes and their associated gene families were then categorized into specific N-cycling processes based on this database.

### 2.6 Statistical analysis

Data analysis was performed using SPSS Statistics (version 26, IBM Corporation, Armonk, NY, USA), employing analysis of variance (ANOVA) to evaluate statistically significant differences among variables. *Post hoc* pairwise comparisons were performed using Duncan’s multiple range test to assess mean differences between individual variables. Statistical significance was defined as *p* < 0.05. Bar charts, histograms, correlation plots, and heat maps were plotted using Excel (Microsoft Excel 2021; Microsoft, Redmond, WA, USA) and Origin 21.0 (Origin 21.0, OriginLab Corp., Northampton, MA, USA).

Alpha diversity was assessed using the Shannon index, calculated with Python scikit-bio package.^1^ Differences among treatments were compared using Tukey’s HSD test and the Kruskal–Wallis *H* test, performed with the “vegan” package (Oksanen, 2017) in R results were visualized using ggplot2 (Zhang R. H. et al., 2025; Zhang Y. Z. et al., 2025). To determine compositional differences, non-metric multi-dimensional scaling (NMDS) was performed based on Bray–Curtis dissimilarities, also using the “vegan” package, and visualized with ggplot2. Microbial species annotation was performed at different taxonomic levels using the Kaiju software (Menzel et al., 2016) against the Nr database. Abundances of dominant bacterial and fungal phyla were visualized in circular layouts using Circos (version 0.69-3, http://circos.ca/) (Krzywinski et al., 2009).

Functional annotation was conducted using the Kyoto Encyclopedia of Genes and Genomes (KEGG) database v87.1 and N cycling-related genes were selected to calculate their abundance in each sample. Heatmaps of the N-cycling genes were created using the pheatmap package in R, while their relative abundance was visualized using ggplot2 (Zhang R. H. et al., 2025; Zhang Y. Z. et al., 2025).

Gene correlation networks and bubble charts depicting the functional classification of genes were analyzed based on the relative abundance of N-cycling genes and visualized using Origin 21.0 (OriginLab Corp., Northampton, MA, USA). Random forest modeling was employed to assess the relative influence of soil environmental factors, nitrogen (N) transformation rates, microbial communities, and N-acquisition-related genes on soil available nitrogen (AN). Variable importance was determined by the percentage increase in the mean squared error (%IncMSE); higher values indicate greater significance. The significance of the models and cross-validated *R*^2^ values were assessed with 1,000 permutations of the response variable, using the “A3” package (Fortmann-Roe, 2015). Similarly, the significance of each predictor for the response variables was assessed with the “rfPermute” package (Archer, 2022).

Structural equation modeling (SEM) was employed to analyze the direct and indirect effects of *E. jolkinii* invasion on soil environmental factors, N-transformation rates, microbial populations, and genes involved in N-acquisition, as well as their collective impact on rhizosphere soil N availability. The model was constructed in R using the “plspm” package. The overall predictive performance of the model was evaluated using the pseudo goodness-of-fit index.

## 3 Results

### 3.1 Responses of soil environmental factors and N components to the invasion of *E. jolkinii*

**Figure 3:**
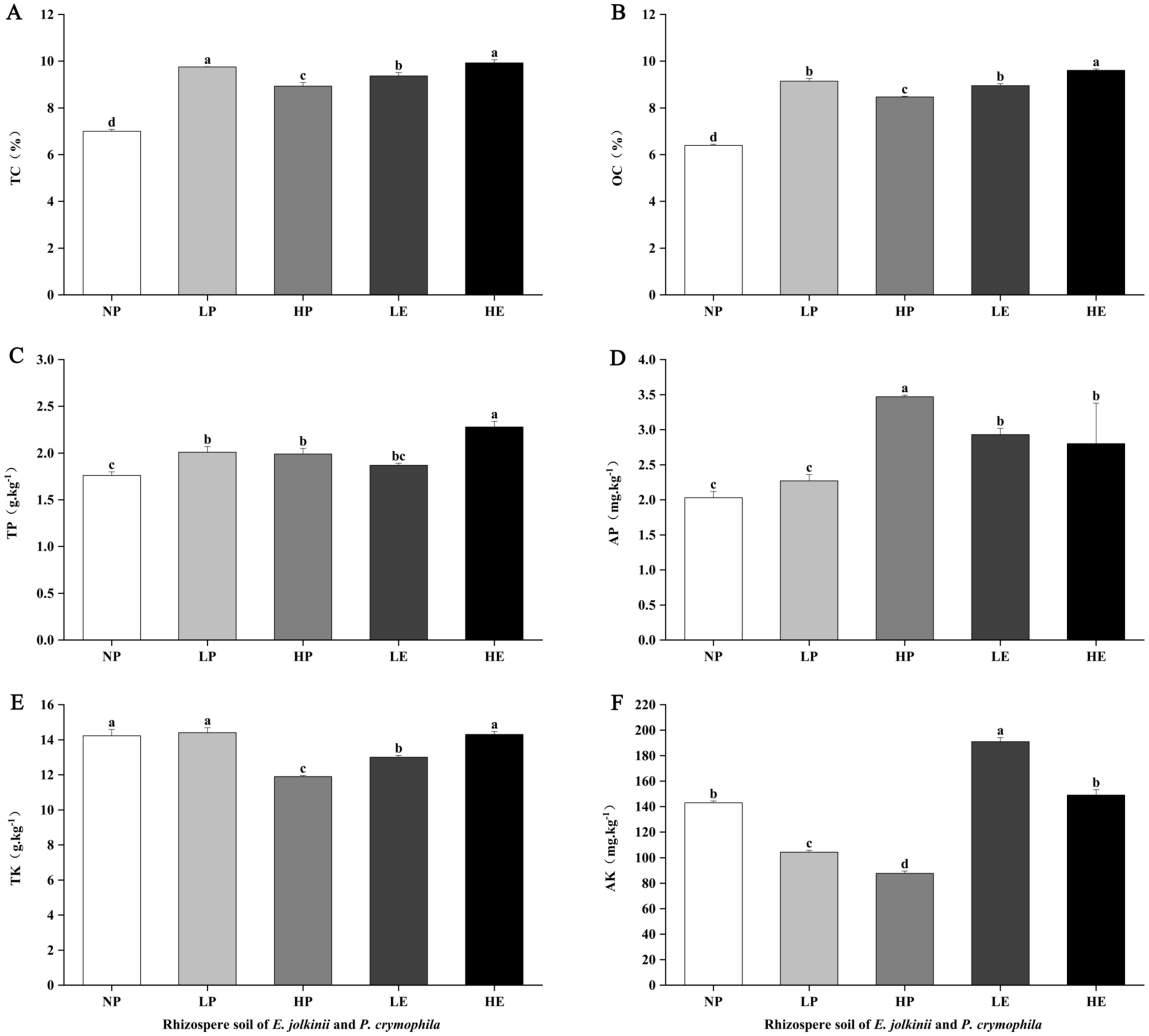
The impact of *E. jolkinii* invasion on soil environmental factors. Changes in total carbon (TC) **(A)**; organic carbon (OC) **(B)**; total phosphorus **(C)**; available phosphorus **(D)**; total potassium (TK) **(E)**, and available potassium (AK) **(F)** of *E. jolkinii* and *P. crymophila* rhizosphere soils (mean ± SE, *n* = 3). Different letters above bar plots indicate significant differences (Duncan test, *p* < 0.05). NP, *P. crymophila* rhizosphere soils of *E. jolkinii* non-invasion patches; LP, *P. crymophila* rhizosphere soils of *E. jolkinii* lightly invaded patches; LE, *E. jolkinii* rhizosphere soils of *E. jolkinii* lightly invaded patches; HP, *P. crymophila* rhizosphere soils of *E. jolkinii* heavily invaded patches; HE, *E. jolkinii* rhizosphere soils of *E. jolkinii* heavily invaded patches.

The invasion of *E. jolkinii* can cause significant changes in soil environmental factors and N components. Comparative analysis of these parameters in the rhizosphere soils of *P. crymophila* across different patches revealed that the expansion of *E. jolkinii* significantly altered soil environmental factors and N components in the rhizosphere of *P. crymophila* (Figures 2, 3). Specifically, the invasion of *E. jolkinii* reduced TK, AK, and N components (HN, NO_3_^−^–N, NH_4_^+^–N, MBN), as well as soil pH in the rhizosphere soil of *P. crymophila*. Conversely, the contents of TN, TP, and AP increased significantly (*p* < 0.05) with the increasing invasion intensity of *E. jolkinii* (Figures 2, 3), whereas TC and OC exhibited an initial increase followed by a decline as the invasion progressed. Further comparisons of soil environmental factors and N components in the rhizosphere of *E. jolkinii* across patches with varying invasion levels revealed that, except for HN, AK, and AP, all other environmental factors and N components were significantly higher in the rhizosphere soil of HE than in that of LE (*p* < 0.05). This result indicates that as *E. jolkinii* expands, its ability to accumulate nutrients significantly increases. Notably, the content of soil NO_3_^−^–N and NH_4_^+^–N increased by 30.78% and 18.71%, respectively.

Comparing the environmental factors and N components in the rhizosphere soil of *E. jolkinii* and *P. crymophila* within the same patch revealed that, as the coverage of *E. jolkinii* increased, its rhizosphere soil exhibited significantly greater nutrient enrichment than that of *P. crymophila* (Figures 2, 3). In lightly invaded patches, the contents of N components, AP and AK in the rhizosphere soil of *E. jolkinii* were significantly higher than those in the rhizosphere soil of *P. crymophila* (*p* < 0.05). In heavily invaded patches, all measured environmental factors and N components, except for AP, were significantly higher in the rhizosphere soil of *E. jolkinii* than in that of *P. crymophila*. This enrichment was particularly pronounced for soil N components, including TN, HN, NH_4_^+^–N, NO_3_^−^–N, and MBN (*p* < 0.05; Figure 2). In heavily invaded patches, the contents of HN, NO_3_^−^–N, and NH_4_^+^–N of *E. jolkinii* rhizosphere soil increased by 1.45, 6.73, and 2.72 times, respectively, compared to those of *P. crymophila.*

### 3.2 Responses of soil N-transformation rates to the invasion of *E. jolkinii*

**Figure 4:**
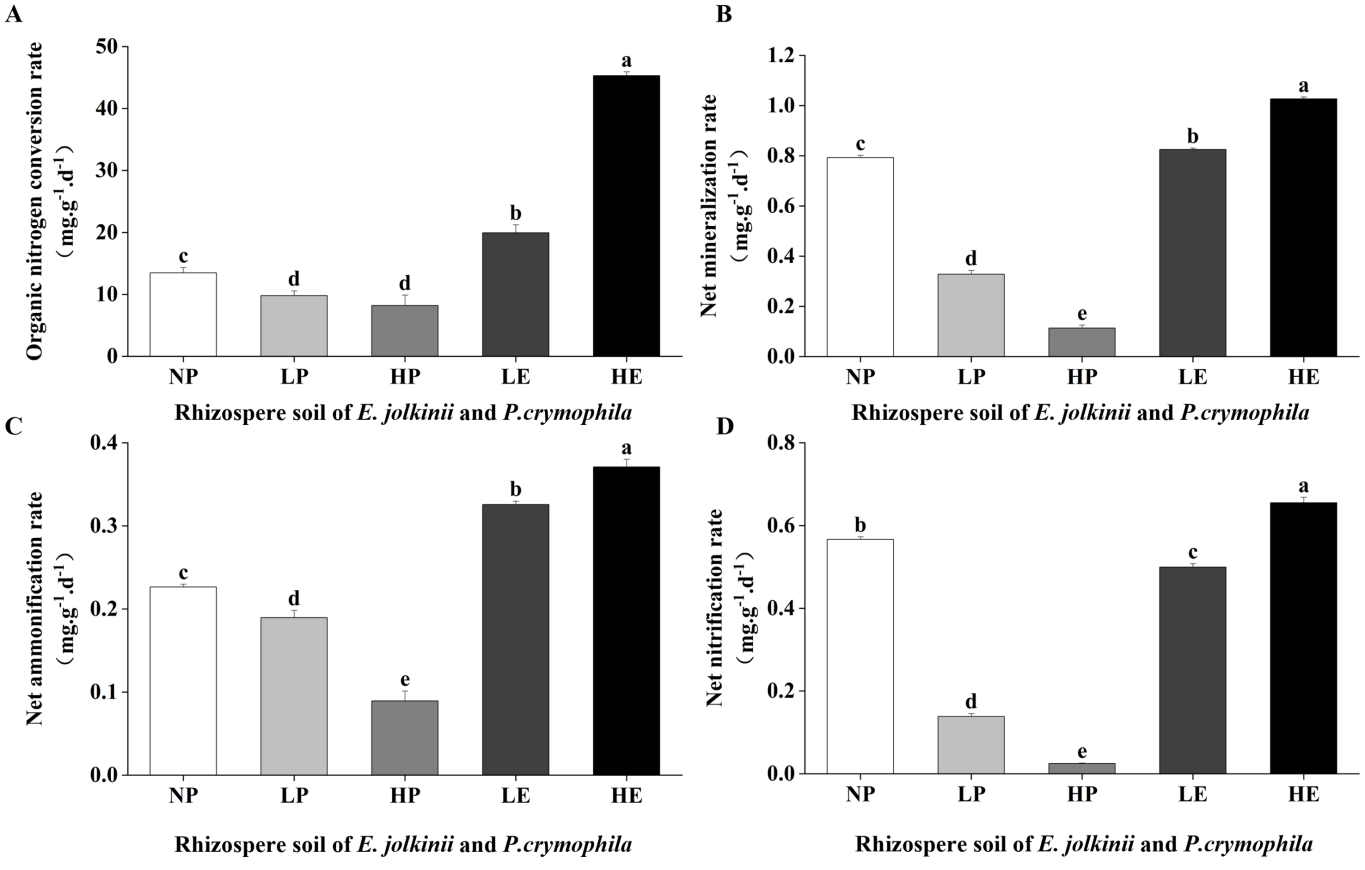
The impact of *E. jolkinii* invasion on soil nitrogen (N) transformation rates. Changes in organic nitrogen conversion rate (ONCR) **(A)**; net mineralization rate (MR) **(B)**; net ammonification rate (AR) **(C)**; and net nitrification rate (NR) **(D)** of *E. jolkinii* and *P. crymophila* rhizosphere soils (mean ± SE, *n* = 3). Different letters above bar plots indicate significant differences (Duncan test, *p* < 0.05). NP, *P. crymophila* rhizosphere soils of *E. jolkinii* non-invasion patches; LP, *P. crymophila* rhizosphere soils of *E. jolkinii* lightly invaded patches; LE, *E. jolkinii* rhizosphere soils of *E. jolkinii* lightly invaded patches; HP, *P. crymophila* rhizosphere soils of *E. jolkinii* heavily invaded patches; HE, *E. jolkinii* rhizosphere soils of *E. jolkinii* heavily invaded patches.

The invasion of *E. jolkinii* led to significant changes in the N transformation rates in the rhizosphere soil of the two species. Specifically, as the invasion intensity of *E. jolkinii* increased, the N transformation rates in the rhizosphere soil of *P. crymophila* decreased significantly (*p* < 0.05). Conversely, these rates were significantly increased in the rhizosphere soil of *E. jolkinii* (Figure 4). Additionally, within the same patch, the rhizosphere soil of *E. jolkinii* (in both lightly and heavily invaded patches) exhibited significantly higher rates of ONCR, MR, AR, and NR compared to that of *P. crymophila* (*p* < 0.05). Collectively, these findings indicate that *E. jolkinii* invasion substantially modified N transformation rates in the rhizosphere soils of both *P. crymophila* and itself, potentially serving as a critical factor influencing soil N accumulation.

### 3.3 Responses of soil microbial diversity and compositions to the invasion of *E. jolkinii*

**Figure 5:**
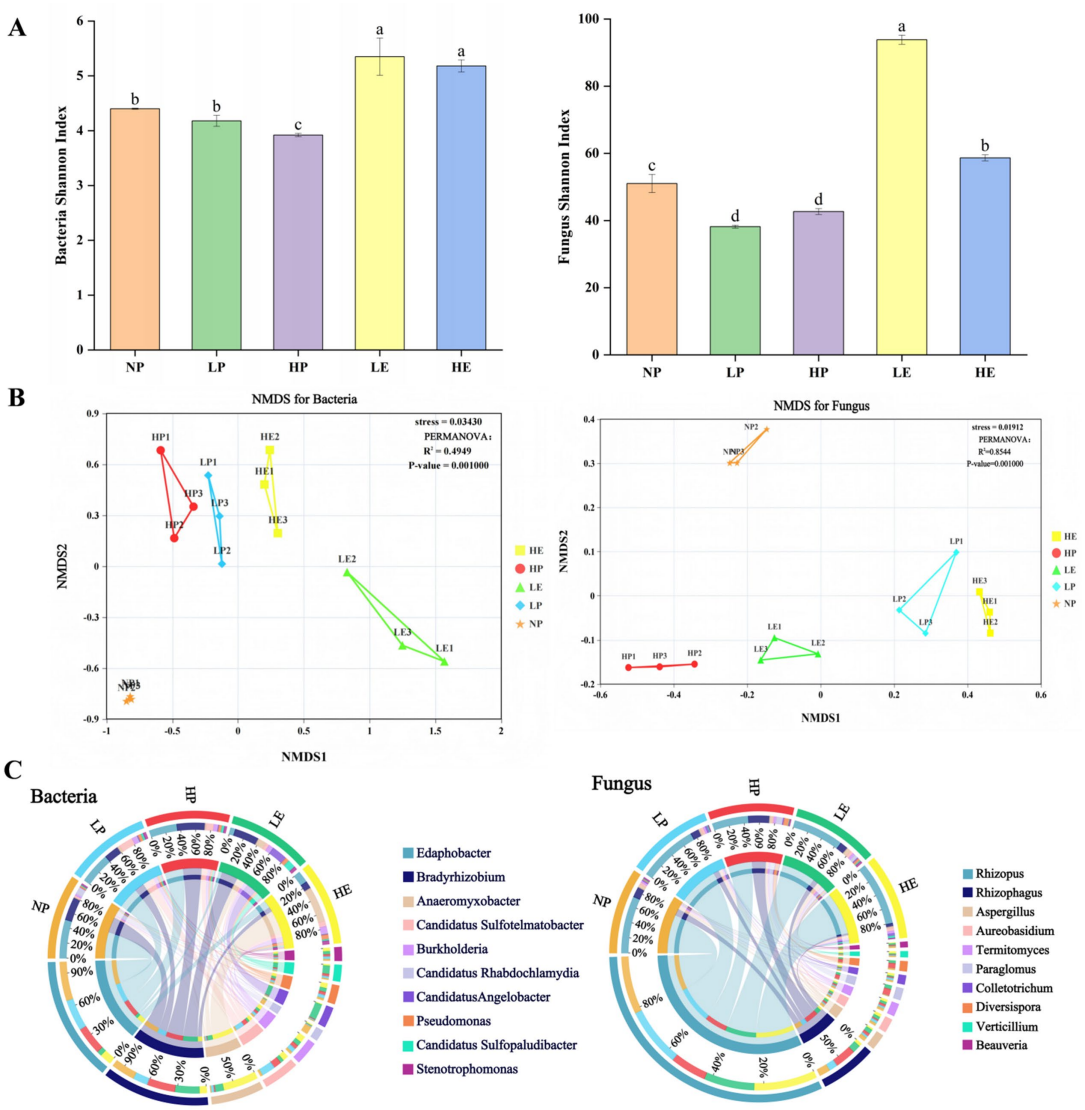
The impact of *E. jolkinii* invasion on microbial diversity patterns and community traits. **(A)** Bar plots of Shannon index of bacteria and fungi of *E. jolkinii* and *P. crymophila* rhizosphere soils. The letters above the boxes indicate significant differences between treatments (*p* < 0.05, *n* = 3). **(B)** NMDS plots of the first two principal components based on the bacterial and fungal community composition of *E. jolkinii* and *P. crymophila* rhizosphere soils, according to the Bray–Curtis distance metrics and PERMANOVA. **(C)** Circos plots of the dominant genera of bacterial and fungal communities of *E. jolkinii* and *P. crymophila* rhizosphere soils. NP, *P. crymophila* rhizosphere soils of *E. jolkinii* non-invasion patches; LP, *P. crymophila* rhizosphere soils of *E. jolkinii* lightly invaded patches; LE, *E. jolkinii* rhizosphere soils of *E. jolkinii* lightly invaded patches; HP, *P. crymophila* rhizosphere soils of *E. jolkinii* heavily invaded patches; HE, *E. jolkinii* rhizosphere soils of *E. jolkinii* heavily invaded patches. The thickness of each ribbon represents the abundance of each taxon. The tick above the outer segment indicates the normalized relative abundance of each taxon.

Our results indicated that the rhizosphere soil microbial community of both plant species changed significantly in response to the invasion of *E. jolkinii*. Specifically, the expansion of *E. jolkinii* notably decreased the Shannon index of both bacteria and fungi in the rhizosphere soil of *P. crymophila*. Within the same patch, the bacterial and fungal Shannon indices were significantly higher in the rhizosphere soil of *E. jolkinii* than in that of *P. crymophila* (Figure 5A). Furthermore, NMDS-based PERMANOVA analysis confirmed significant differences in the community structure of bacteria (*p* = 0.001) and fungi (*p* = 0.001) between the rhizosphere soil of both *P. crymophila* and *E. jolkinii*. The invasion of *E. jolkinii* considerably influenced the bacterial and fungal community structures in the rhizosphere soil of *P. crymophila* (Figure 5B; Supplementary methods).

The invasion of *E. jolkinii* induced significant changes in the microbial communities within the rhizosphere soil of the two species. A comparative analysis across different patches revealed that the expansion of *E. jolkinii* significantly altered the genus-level composition of both bacterial and fungal communities in the rhizosphere soil of *P. crymophila.* Specifically, the invasion of *E. jolkinii* decreased the relative abundance of several taxa in the *P. crymophila* rhizosphere soil, including the bacteria Edaphobacter, Candidatus Rhabdochlamydia, and the fungi Rhizopus, Aspergillus, Aureobasidium, Colletotrichum, and Verticillium. Conversely, the relative abundance of other microbes, such as the bacteria Bradyrhizobium, Candidatus Angelobacter, Candidatus Sulfopaludibacter, and Stenotrophomonas, and the fungi Rhizophagus, Paraglomus, and Beauveria increased with increasing invasion intensity of *E. jolkinii* (Figure 5C). Whereas the bacteria Anaeromyxobacter, Candidatus Sulfotelmatobacter, Burkholderia, and Pseudomonas exhibited an initial increase followed by a decline as the invasion progressed. Further analysis of the *E. jolkinii* rhizosphere soil across patches with varying invasion levels showed that the relative abundance of most microbial taxa was significantly greater in HE compared to LE. Exceptions to this trend included the bacteria Bradyrhizobium, Candidatus Angelobacter, and Candidatus Sulfopaludibacter, and the fungi Rhizophagus, Aspergillus, Paraglomus, and Diversispora. This result underscores that the expansion of *E. jolkinii* enhances its ability to alter microbial communities.

A comparison of the rhizosphere microbial communities between *E. jolkinii* and *P. crymophila* within the same patch showed that as *E. jolkinii* expansion, its rhizosphere soil became significantly more enriched in both bacteria and fungi compared to that of *P. crymophila* (Figure 5C). Furthermore, when comparing the relative microbial abundance between the two species within the same patch, all bacterial and fungal taxa, except for Edaphobacter (bacteria), Candidatus Sulfopaludibacter (bacteria), Rhizophagus (fungus), and Paraglomus (fungus), showed significantly higher levels in the rhizosphere soil of *E. jolkinii* (in both lightly and heavily invaded patches) than in that of *P. crymophila* (*p* < 0.05). Collectively, these findings indicate that *E. jolkinii* invasion substantially modifies the bacterial and fungal composition in the rhizosphere soil of both *P. crymophila* and itself, which may play a key role in affecting soil nitrogen transformation.

### 3.4 Responses of soil N-cycling genes to the invasion of *E. jolkinii*

**Figure 6:**
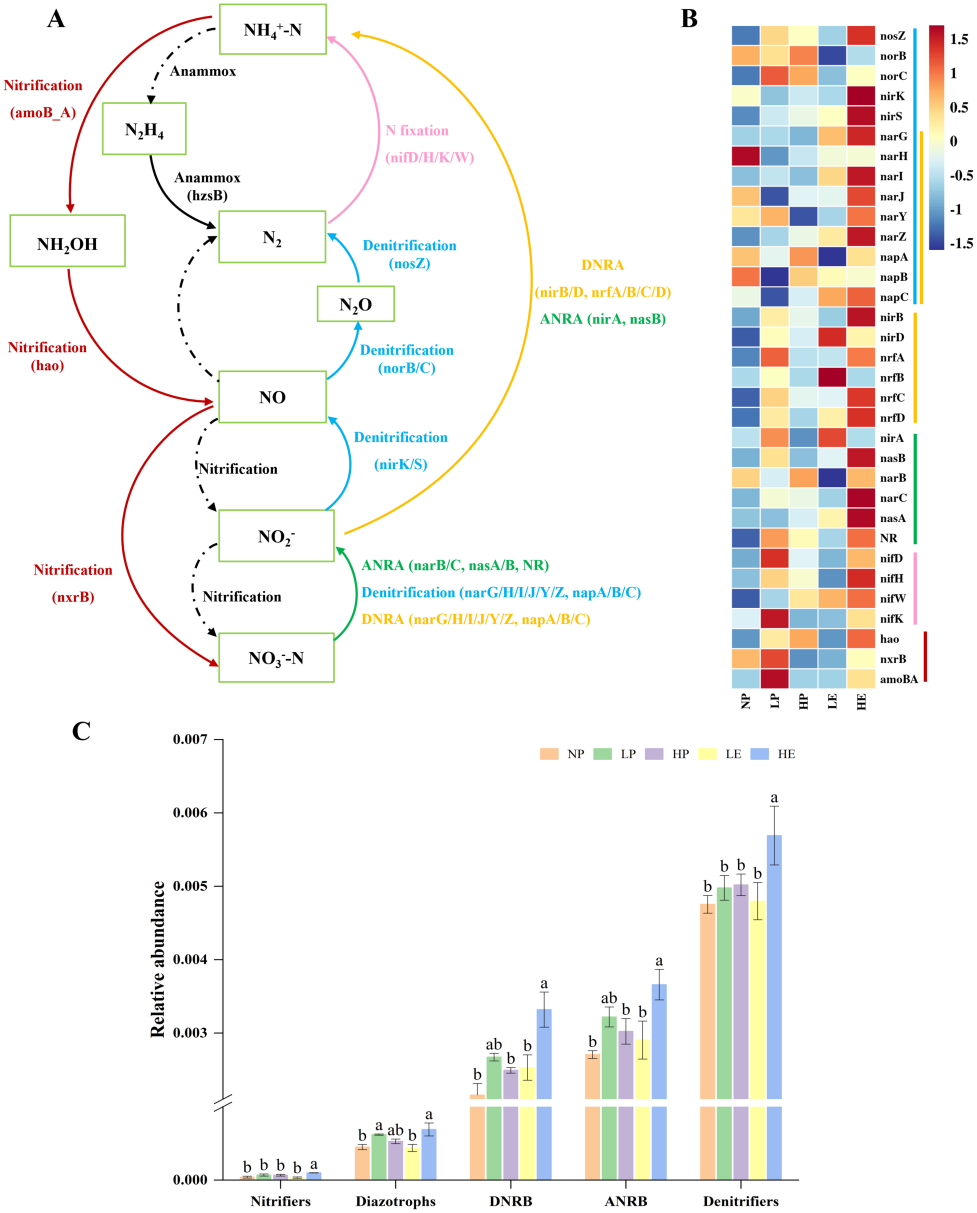
Key N transformation processes and corresponding functional genes **(A)**, the changes in abundance of these genes in different groups **(B)**, and the abundance of microbial populations corresponding to key functional genes **(C)**. NP, *P. crymophila* rhizosphere soils of *E. jolkinii* non-invasion patches; LP, *P. crymophila* rhizosphere soils of *E. jolkinii* lightly invaded patches; LE, *E. jolkinii* rhizosphere soils of *E. jolkinii* lightly invaded patches; HP, *P. crymophila* rhizosphere soils of *E. jolkinii* heavily invaded patches; HE, *E. jolkinii* rhizosphere soils of *E. jolkinii* heavily invaded patches.

To identify the predominant microorganisms involved in soil N-cycling, such as diazotrophs, nitrifiers, denitrifiers, assimilatory nitrate-reducing bacteria (ANRB), and dissimilatory nitrate-reducing bacteria (DNRB), a total of 33 biomarker genes were analyzed. These genes belong to 5 key N-cycling processes: nitrification, denitrification, N fixation, assimilatory nitrate reduction to ammonium (ANRA) and dissimilatory nitrate reduction to ammonium (DNRA) (Figure 6A). The abundances of these functional genes, along with their associated microbial communities, varied significantly across different treatments in the rhizosphere soil (Figures 6B,C). Specifically, light invasion by *E. jolkinii* stimulated the enrichment of N-cycling microorganisms and the expression of their functional genes in the rhizosphere soil of *P. crymophila*. Conversely, further expansion of *E. jolkinii* inhibited both the abundance and gene expression of microorganisms in the rhizosphere soil of *P. crymophila*. Additionally, the invasion of *E. jolkinii* significantly promoted the accumulation of N-cycling microorganisms and the expression of their genes in its own rhizosphere soil, with the highest gene abundance observed in HE (Figures 6B,C).

The abundances of all microbial groups involved in N transformation in the rhizospheres of *E. jolkinii* and *P. crymophila* in non-invaded patches, lightly invaded, and heavily invaded patches were further compared. Figure 6C illustrates that the invasion of *E. jolkinii* significantly affected the populations of diazotrophs, nitrifiers, denitrifiers, ANRB and DNRB. In the lightly invaded patches, the abundance of various N-cycling microorganisms of *P. crymophila* soil was higher than that of *E. jolkinii* and non-invaded *P. crymophila* soils, with particularly pronounced increases in diazotrophs, DNRB, and ANRB. However, in heavily invaded patches, the total abundance of microorganisms in *E. jolkinii* rhizosphere soil was significantly higher than that in *P. crymophila* rhizosphere soil. These findings indicate that as the invasion intensity of *E. jolkinii* increased, disturbance to the N-cycling microbial communities associated with itself and companion plant gradually intensified.

### 3.5 Taxonomic distribution of key functional genes in relation to the invasion of *E. jolkinii*

**Figure 7:**
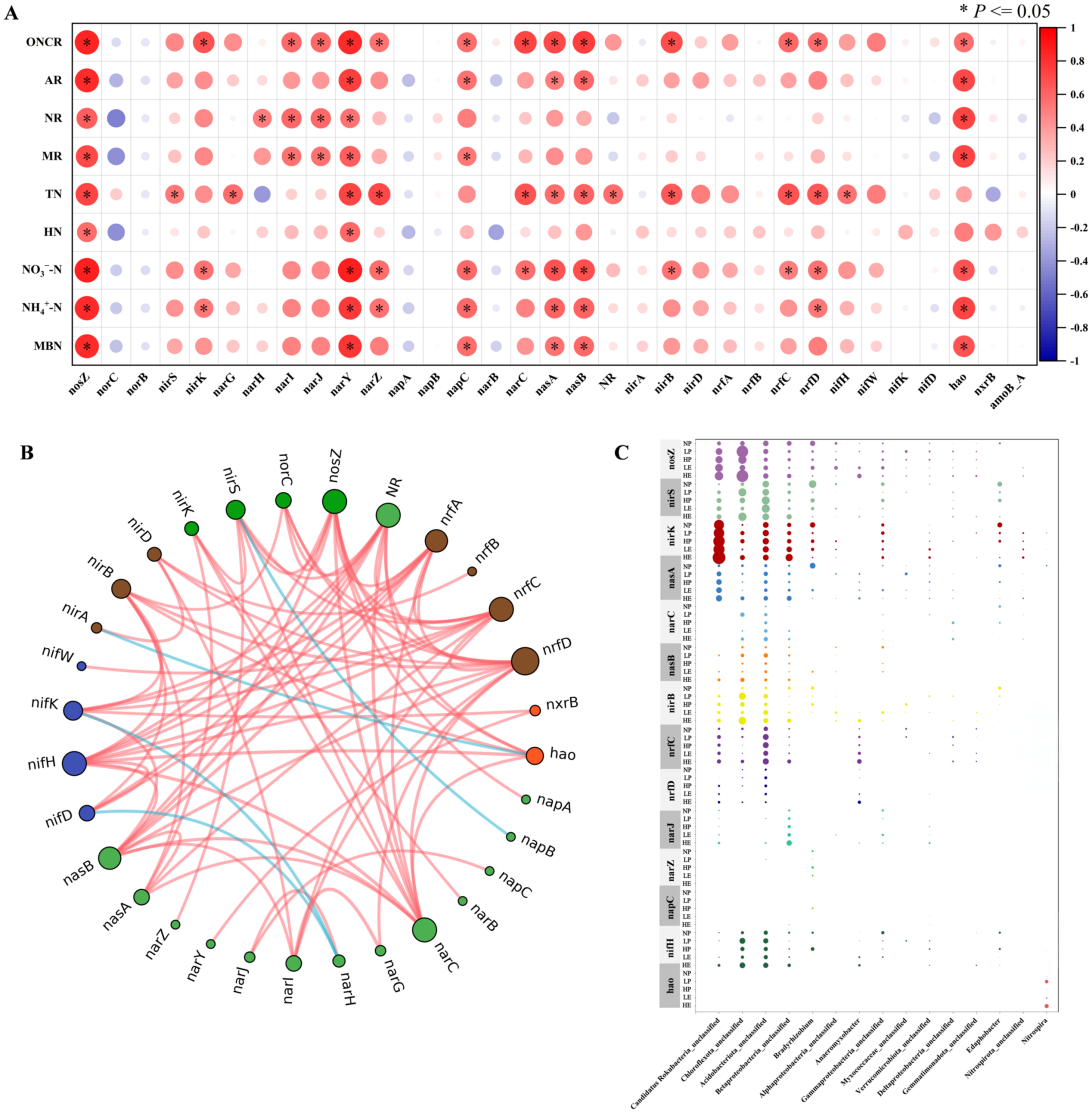
Correlation heatmap **(A)**, interaction network graph based on functional gene data correlation **(B)**, and the taxonomic distribution (phylum level) of 15 key functional genes **(C)** (*nosZ*, *nirS*, *nirK*, *nasA*, *narC*, *nasB*, *nirB*, *nrfC*, *nrfD*, *narJ*, *narZ*, *narY*, *napC*, *nifH*, and *hao*) and bubble plots showing the variation in the abundance at the genera level for each functional gene under different groups.

The 33 functional genes identified through the metagenomic analysis were further analyzed. The distribution and relative abundance of soil N cycling functional genes were associated with soil N transformation rates and the accumulation of AN. Correlation analysis indicated that 15 genes, including nosZ, nirS, nirK, and nasA, showed a significant positive correlation with soil AN component (HN, NO_3_^−^–N, and NH_4_^+^–N) and soil N transformation rates (Figure 7A). Furthermore, a functional gene interaction network revealed that genes such as *nosZ*, *nirS*, *nirK*, and *nasA* interacted with multiple functional genes (Figure 7B). Therefore, we selected 15 genes for further analysis of their taxonomic distribution at the genus level. These genes—*nosZ*, *nirS*, *nirK*, *nasA*, *narC*, *nasB*, *nirB*, *nrfC*, *nrfD*, *narJ*, *narZ*, *narY*, *napC*, *nifH*, and *hao* are involved in key N cycling processes, including nitrification, denitrification, nitrogen fixation, nitrate reduction, assimilation of nitrate reduction to ammonium (ANRA) and dissimilatory nitrite reduction to ammonium (DNRA).

Following taxonomic assignment, these 15 functional genes were distributed across 15 genera (Figure 7C). Most genes were primarily distributed at the genera level in Candidatus Rokubacteria_unclassified, Chloroflexota_unclassified, Acidobacteriota_unclassified, and Gammaproteobacteria_unclassified. For example, *nosZ*, *nirS*, *nirK*, *nasA*, *nirB*, *nrfC*, and *nifH* were detected in most genera. *narC*, *nasB*, and *nrfD* were mainly detected in Chloroflexota_unclassified, Acidobacteriota_unclassified, and Betaproteobacteria_unclassified. *narJ* was distributed in Candidatus Rokubacteria_unclassified, Acidobacteriota_unclassified, Betaproteobacteria__unclassified, and Gammaproteobacteria_unclassified. In contrast, *narZ*, *napC*, and *hao* were distributed in only one genus. Notably, some genes were detected in only three genera, whereas others were noted in several genera, highlighting the diversity in the taxonomic distribution among these N-cycling functional genes.

Further analysis of the differences in gene enrichment at the genus level revealed that, within the same patch, the abundance of most genes in the rhizosphere soil of *P. crymophila* increased initially and then decreased with increasing *E. jolkinii* invasion intensity. In contrast, most genes in the rhizosphere soil of *E. jolkinii* increased significantly with increasing invasion intensity. When comparing gene enrichment across different genera between the rhizosphere soils of *E. jolkinii* and *P. crymophila* within the same patch, most genes showed no significant differences in lightly invaded patches. However, in heavily invaded patches, the gene enrichment in the rhizosphere soil of *E. jolkinii* was significantly higher than that in the rhizosphere soil of *P. crymophila*. Moreover, significant differences in functional microbial community composition were observed across groups, suggesting that the invasion and expansion of *E. jolkinii* may regulate the abundance and distribution of key N-cycling microorganisms.

### 3.6 Direct and indirect pathways through which *E. jolkinii* promotes the accumulation of AN

**Figure 8:**
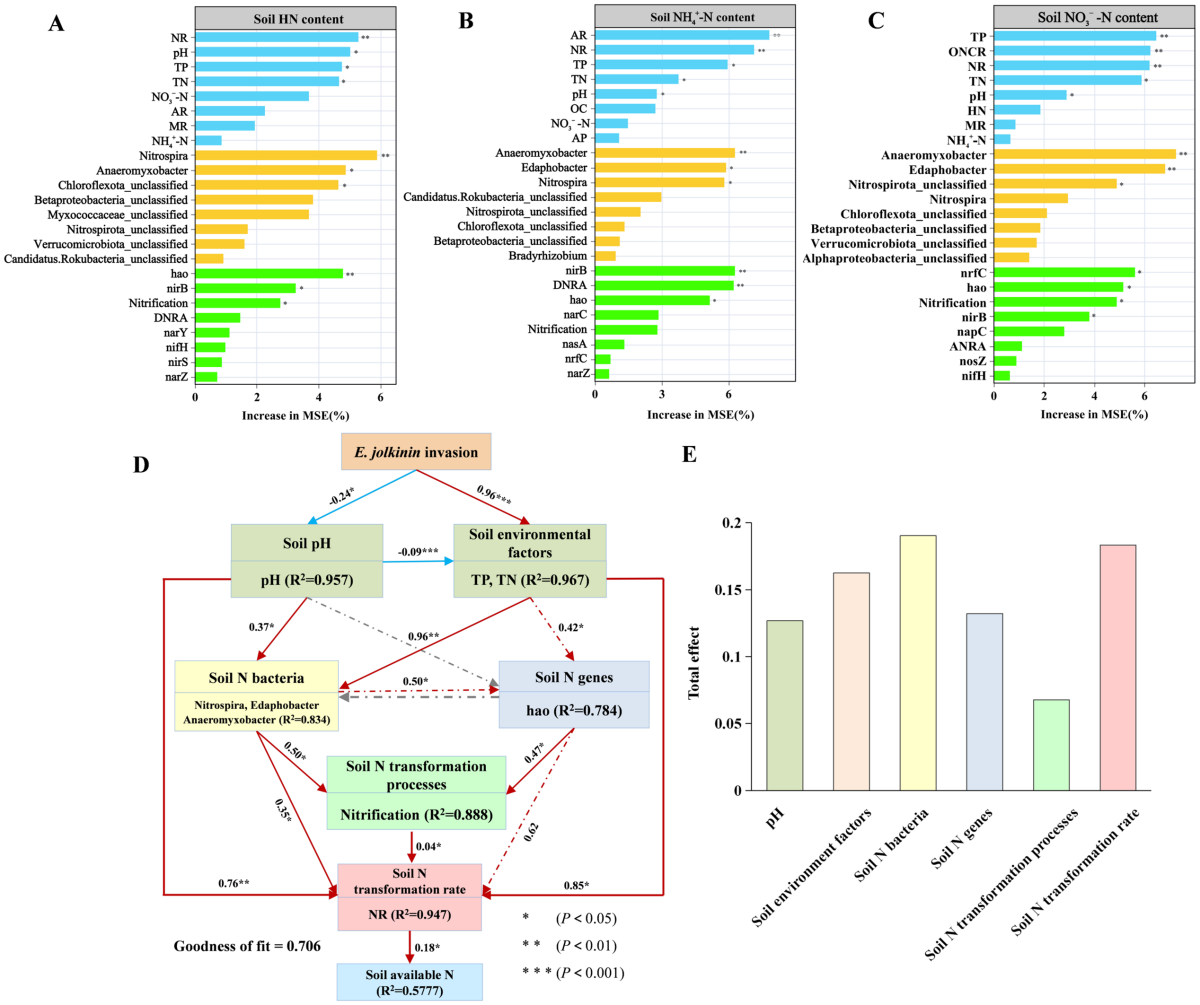
Random forest predictor of the importance of the soil environment, N transformation rate, N-acquiring functional genes, and functional groups as the drivers of soil AN component **(A–C)**. **(D,E)** Structural equation model describing the effects and pathways of multiple drivers, including the soil environment, the N transformation rates, and key N-acquiring gene (*hao*) abundance selected based on random forest analyses, on the soil available N components. Blue and red arrows indicate the negative and positive effects, respectively. Numbers on arrows are standardized path coefficients. *R*^2^ indicates the proportion of variance explained **(A,C)**. Standardized total effects of each individual driver on AN **(B,D)**. *, **, and *** indicate significance at probability levels of <0.05, <0.01, and <0.001, respectively.

The results of random forests indicated that soil N genes, soil environmental factors, and N-acquiring microbial community composition served as reliable predictors of soil N availability (Figures 8A–C). Among the environmental factors, TP, TN and pH were the primary predictors of AN. Additionally, the abundance of *hao* and microbial genera such as Nitrospira, Edaphobacter, and Anaeromyxobacter were significant predictors of AN. Consequently, among the various N-cycling pathways, nitrification may be the key processes predicting AN dynamics (Figures 8A–C).

Further analysis using the SEM model revealed that the NR exerts a potent and direct effect on the soil AN content. This effect was primarily regulated by soil pH, TP, and TN and the abundances of nitrification-related functional genes (*hao*) and N-acquiring microorganisms (Nitrospira, Edaphobacter, and Anaeromyxobacter) (goodness of fit: 0.706) (Figure 8D). Specifically, the invasion of *E. jolkinii* significantly and negatively regulated soil pH (path coefficient: −0.24, *p* < 0.05), while significantly and positively regulating soil environmental factors (TP and TN) (path coefficient: 0.96, *p* < 0.001). The soil pH significantly and negatively regulated soil environmental factors (TP and TN) (path coefficient: −0.09, *p* < 0.001). The soil pH and environmental factors significantly and positively regulated the soil transformation rate (path coefficient: 0.76, *p* < 0.01; 0.85, *p* < 0.05, respectively), thereby mediating the accumulation of bioavailable N. Additionally, the soil pH and environmental factors positively influenced.

N-acquiring microbial populations (path coefficients: 0.37, *p* < 0.05; 0.96, *p* < 0.01, respectively). The soil environmental factors also positively influenced N-related genes (path coefficient: 0.42, *p* < 0.05). In turn, both the N-acquiring microbial populations and the N-related genes positively regulated the soil N transformation process (path coefficients: 0.50, *p* < 0.05; 0.47, *p* < 0.05, respectively). Furthermore, they positively regulated the soil N transformation rate (path coefficient: 0.35, *p* < 0.05; 0.62, *p* > 0.05, respectively) and the AN content (path coefficient: 0.18, *p* < 0.05). Therefore, N-acquiring microbial populations exerted the most positive composite influence on the soil AN content (Figure 8E).

## 4 Discussion

### 4.1 Rhizosphere control by *E. jolkinii* on soil N transformations

Invasive plants expand their distribution by altering the nutrient balance of native vegetation and changing soil properties. Furthermore, invasive species exhibit a greater competitive advantage over native plants in N acquisition. In this study, we found that the invasion of *E. jolkinii* reduced the pH of the rhizosphere soil of companion plant while increasing the pH of its own rhizosphere soil. These findings are consistent with the results of Yuan C. Z. et al. (2024), who demonstrated that the invasive plant *Chromolaena odorata* significantly altered soil pH in tropical coral islands, establishing favorable conditions for soil nitrification. In addition, we demonstrated that the invasion of *E. jolkinii* affected N transformation rates in the rhizosphere soil. Notably, invasive species can enhance rhizosphere nutrient cycling and mitigate N losses by regulating N transformation rates, thereby enhancing N availability in the root-zone (Yuan L. et al., 2024). This modulation of soil N change is closely linked to the competitive interactions between invasive and native dominant species, particularly regarding their resource acquisition strategies (Insu et al., 2017). In the present study, the contents of AN (NO_3_^−^–N, NH_4_^+^–N, and HN) in *E. jolkinii* rhizosphere soils were considerably higher than those in *P. crymophila*. This difference may be attributed to the higher N transformation rates of *E. jolkinii* compared to companion plant with interspecific competition. Moreover, invasive plants alter environmental factors, such as pH, in the rhizosphere, thereby affecting the supply and fluctuations of N in the rhizosphere and influencing N transformation rates (Insu et al., 2017). For example, *Stellera chamaejasme* root exudates synthesize a variety of alkaloids that not only modify soil pH but also influence N accumulation and reshape microbial communities (Zhang R. H. et al., 2025; Zhang Y. Z. et al., 2025). In addition to directly regulating N transformation, invasive plants can also alter the N-acquiring microbial communities, thereby influencing microbially driven rhizosphere N accumulation (Fan et al., 2025). Therefore, additional research is required to identify key constituents of N-acquiring microbiomes within N transformation.

### 4.2 Rhizosphere control by *E. jolkinii* on soil N- acquisition microbial communities and function genes

Rhizosphere N conversion is driven by soil microorganisms (Wu et al., 2025; Xu et al., 2025). Yuan L. et al. (2024) demonstrated that the invasion of poisonous weeds can enhance the abundance and functionality of soil microorganisms and enzymatic processes. Consistently, our field evidence revealed that the invasion of *E. jolkinii* affected the composition of microbial communities associated with N-acquiring. This finding is also consistent with previous studies demonstrating that the spread of invasive poisonous weeds can alter the rhizosphere N-cycling microbiomes (Morgan Luce et al., 2016; Wang et al., 2018).

Previous research has primarily concentrated on the individual functional genes involved in nitrification, denitrification, and N fixation in invasive plants or toxic weeds. These studies have particularly focused on the abundance of marker genes such as nifH (N fixation), amoA (ammonia oxidation), nirK and nirS (denitrification) (Li et al., 2024; Yuan C. Z. et al., 2024). In contrast, our study provides a comprehensive analysis of the abundance of functional genes governing soil N enrichment to evaluate the impact of *E. jolkinii* invasion on key biogeochemical processes. We found that *E. jolkinii* invasion significantly affected several critical N-cycling pathways, including denitrification, N fixation, ANRA, nitrification, and DNRA. These findings underscore the potential ecological consequences of *E. jolkinii* invasion on soil N transformations and associated microbial communities. However, it should be noted that the sampling sites of this study were limited to the enclosed area of the Shangri-La National Grassland Monitoring Station, and only a single sampling period was conducted. These factors may constrain the generalizability of the findings across different soil types, climatic conditions, and temporal stages of *E. jolkinii* invasion. Therefore, our research team is currently conducting comparative studies on the effects of *E. jolkinii* invasion on soil N transformation across various regions and time frames. This aims to further validate the conclusions drawn in this study and to elucidate the impacts of *E. jolkinii* invasion on soil N transformation from both spatial and temporal dimensions.

As a fundamental process in N transformation pathways within terrestrial ecosystems, soil nitrification plays an integral role in regulating the availability and cycling of reactive N species (Hu et al., 2025). Nitrification is catalyzed by chemolithotrophic microorganisms, specifically ammonia-oxidizing bacteria and nitrite-oxidizing bacteria, which sequentially oxidize NH_4_^+^–N to NO_3_^−^–N through the nitrification cycle (Wang et al., 2024). Our results demonstrated that the rhizosphere soils of LP and HE exhibited a marked increase in the abundance of functional genes *hao*, *nxrB*, and *amo_B*, which are associated with nitrification processes. Furthermore, the abundance of hao exhibited a strong correlation with soil N content, aligning with the results of Xu et al. (2024). Our analysis of the distribution of *hao* at the genus level revealed an increased abundance of Nitrospira. Daims et al. (2015) reported that bacteria from the genus Nitrospira within the phylum Nitrospirota can complete ammonia oxidation, directly converting ammonia into nitrate. This highlights the essential role of Nitrospira microorganisms in the soil N cycle. Thus, one of the primary pathways for *E. jolkinii* to achieve invasion and expansion is by optimizing N utilization efficiency and reducing N loss through regulation of the abundance of Nitrospira microorganisms and associated functional genes. This finding is also consistent with that of Yu et al. (2020), who discovered that nitrification is the main factor promoting the accumulation of effective N in the rhizosphere of the invasive plant *Mikania micrantha*.

In the process of ANRA and DNRA, NO_3_^−^–N is transformed into NO_2_^−^–N and NH_4_^+^–N. Notably, in HE, the abundance of biomarkers indicative of ANRB and DNRB, including the *nirB/D*, *narB/C*, *nasA/B*, *NR*, *napA/B/C*, *nrfA/B/C/D*, and *narG/H/I/J/Y/Z* genes, was significantly higher than that in the other groups. We also found that the key genes involved in ANRA and DNRA were primarily concentrated within the genus Chloroflexota_unclassified, Acidobacteriota_unclassified, Betaproteobacteria__unclassified, and Anaeromyxobacter. Among them, Chloroflexota_unclassified, Acidobacteriota_unclassified, and Betaproteobacteria__unclassified belong to the phyla Chloroflexota, Acidobacteriota, and Pseudomonadota, respectively. Acidobacteriota possess significant capacity for N fixation and exhibit notable potential in suppressing harmful pathogens through biological mechanisms. These microorganisms play a crucial role in maintaining the equilibrium of the root-associated microbiome and nutrient cycling of *E. jolkinii* (Kruczyńska et al., 2023). Chloroflexota bacteria play a significant role in the conversion of inorganic carbon into organic compounds. They are also actively involved in nutrient cycling, particularly through the mobilization of N and P, highlighting their pivotal role in facilitating symbioses among soil microbes and plants (André and Fradinho, 2024). Chen et al. (2021) identified Chloroflexota as a key contributor to organic matter production and nutrient availability, particularly by reducing the activity of bacteria responsible for N loss. In agricultural soils, Pseudomonadota participates in both denitrification (*nirK/S*) and DNRA (*nrfA*), and its metabolic preferences are regulated by carbon sources and redox conditions. Under nutrient-rich conditions, it converts NO_3_^−^ to NH_4_^+^ via DNRA (Zheng X. J. et al., 2024). Anaeromyxobacter, a facultative anaerobe belonging to the order Myxococcales, is abundant in rhizosphere soils and plays a crucial role in DNRA (Tomita et al., 2026). The capability of Anaeromyxobacter to DNRA was detected in soil environments previously, including upland agricultural, crop rhizospheric, grassland, and polar soils (Masuda et al., 2017). Notably, all these microorganisms contribute to the soil N Cycle. Collectively, these findings indicate that these two processes may be the primary pathways by which *E. jolkinii* invasion regulates N transformation in the soil.

Denitrification is a microbial process that reduces nitrate and nitrite to nitric oxide (NO), nitrous oxide (N_2_O), and nitrogen (N_2_), playing a critical role in N-cycling within ecosystems (Yang et al., 2022). In addition, pH significantly affects the abundance of denitrification-related genes, exhibiting a positive correlation with specific functional genes such as *nirK* (Li et al., 2022). Soil physicochemical factors may mediate variations in the abundance of functional genes by altering the community composition and metabolic activity of denitrifying microorganisms. Among the functional genes related to denitrifying bacteria, our analysis revealed that *nosZ*, *nirS*, and *nirK* exhibited the highest abundance in HE. This finding suggests that *E. jolkinii* invasion not only promotes the denitrification process but also significantly enhances the relative abundance of these key denitrification genes (Yu et al., 2020). This may be due to the alterations in the soil physicochemical properties induced by *E. jolkinii* invasion. Although denitrification generally promotes N loss from the soil and reduces the accumulation of AN, the first step of denitrification—where nitrate is reduced to nitrite—provides a greater amount of nitrite for the DNRA process, thus supplying more substrate for the reduction to ammonia. Consequently, this process also indirectly enhances the pool of available nitrogen in the rhizosphere.

As an integral process within the N cycle, biological N fixation involves converting atmospheric N gas (N_2_) into N compounds, such as ammonia, in the soil. This transformation is vital to sustaining life on Earth (Zheng M. H. et al., 2024). In the present study, the effect of *E. jolkinii* at varying levels of invasion on the N fixation-related genes did not reach statistical significance. However, it decreased the gene abundances of *nifK* and *nifD* in LE, which were primarily affected by the alterations in soil properties induced by the invasion of *E. jolkinii.*

While these findings provide insights and fresh perspectives on how the invasion of *E. jolkinii* affected soil N transformation processes, the results were all based on the relative abundance of functional genes associated with N transformation, which lacked direct evidence regarding gene expression or enzyme activity. In the following phase, we will conduct qPCR validation of these functional genes along with soil microbial activity cultivation experiments to further clarify the mechanisms through which these genes and microorganisms influence soil N transformation processes.

### 4.3 *Euphorbia jolkinii* invasion regulates microbe-driven N accumulation

N-acquiring microbiomes play a significant role in N-cycling and can influence the AN in soils through both direct (N fixation) and indirect (organic anion activated) mechanisms (Wang et al., 2024). Our results indicated that the *hao* gene and TP were the major factors of soil AN. This is consistent with the findings of Xu et al. (2021), demonstrating that environmental factors and microbial communities jointly regulate the effectiveness of soil AN. Consistent with our original hypothesis, the SEM findings revealed that *hao*-driven nitrification may exert a greater influence on enhancing N availability for *E. jolkinii* than on N fixation. Specifically, nitrification is the primary mechanism that promotes the accumulation of NO_3_^−^–N for *E. jolkinii* invasion and expansion, with *hao* considerably contributing to this process. These findings align with previous research demonstrating that toxic weeds prefer NO_3_^−^–N but not NH_4_^+^–N (Guan et al., 2023; Yuan C. Z. et al., 2024). Overall, these findings indicate that *E. jolkinii* is highly dependent on soil N for growth, rather than atmospheric N obtained through N fixation. This result is consistent with conclusions regarding the microbial mechanisms of invasion by *Mikania micrantha* (Yu et al., 2020).

## 5 Conclusion

Our findings highlighted the critical role of N-cycling in interspecific soil nutrient competition associated with the invasiveness of *E. jolkinii*. We demonstrated that *E. jolkinii*, a native invasive plant of Shangri-La, achieves greater N enrichment in its rhizosphere compared to the native dominant *P. crymophila*. This may be partly attributed to the influence of *E. jolkinii* over the abundance of N-acquiring microbial communities, promoting rhizosphere soil N transformations. These transformations increased the supply capacity of soil AN. *E. jolkinii* enriches its rhizosphere with key N-cycling functional microbes, such as Nitrospirota, Edaphobacter, and Anaeromyxobacter. This enrichment likely enhances N accumulation, particularly via hao gene-mediated nitrification, thereby increasing N availability to support its invasion. However, further studies at the enzymatic and transcriptomic levels are required to validate these proposed microbial functions. These results provide novel insights into how plant invasions are promoted via plant–soil interactions. It is essential to note that these conclusions are primarily based on gene abundance and do not directly quantify functional activity. Further validation of gene function and microbial activity will be needed. Besides, given the context-sensitive nature of these interactions, future research should investigate whether the patterns observed in our study apply to other regions where *E. jolkinii* has become invasive or whether they extend to other non-leguminous invaders. Such studies represent crucial future research opportunities to corroborate and extend our findings.

## Data Availability

The data presented in this study are publicly available. The data can be found here: https://www.ebi.ac.uk/ena/browser/, accession number PRJEB108285 (secondary accession number ERP189147). Further inquiries can be directed to the corresponding author.

## Footnotes

1
https://scikit.bio/docs/latest/diversity.html
