# Supplementary material for: The invasion of Euphorbia jolkinii is mediated through the regulation of nitrogen transformation by functional microbial abundance in rhizosphere soils
Source: Front Microbiol. 2026 Feb 24;17:1757844. doi: 10.3389/fmicb.2026.1757844 (PMC12974137; doi:10.3389/fmicb.2026.1757844)
Supplement: Supplementary file 1 [file Table_1.DOCX]

**Supplementary Tables**

**TABLE S1**

Basic information on sampling units

| Patch Types | Total aboveground biomass (g·DM·m^2^) | Aboveground biomass of  *E. jolkinii* (g·DM·m^2^) | Coverage of  *E. jolkinii* (%) | Vegetation condition |
| --- | --- | --- | --- | --- |
| N | 424.27 | 0 | 0 | No invasion of *E. jolkinii*, with *P. crymophila* as the sole dominant species. |
| L | 544.93 | 72.12 | 8.9 | Invasion of *E. jolkinii* occurs, but *P. crymophila* remains the dominant species. |
| H | 640.76 | 301.45 | 41.2 | The severe invasion of *E. jolkinii* leads to its establishment as the dominant species, while the community status of *P. crymophila* is reduced to a subdominant species. |

**TABLE S2**

Influences of the invasion of *E. jolkinii* on the bacterial and fungal communities based on the Bray-Curtis distance matrix with PERMANOVA test. Significance levels of are as follows **P* < 0.05, ***P* < 0.01, and ****P* < 0.001.

| **Bacterial Community** | **Df** | **Sum Of Sqs** | **R^2^** | **F** | **Pr (>F)** |
| --- | --- | --- | --- | --- | --- |
| Treatment | 4 | 2.14 | 0.49 | 2.45 | 0.001*** |
| Residual | 10 | 2.19 | 0.51 |  |  |
| Total | 14 | 4.33 | 1 |  |  |
|  |  |  |  |  |  |
| **Fungal Community** | **Df** | **Sum Of Sqs** | **R^2^** | **F** | **Pr (>F)** |
| Treatment | 4 | 0.7 | 0.85 | 14.67 | 0.001*** |
| Residual | 10 | 0.12 | 0.15 |  |  |
| Total | 14 | 0.82 | 1 |  |  |
