# Supplementary material for: The invasion of Euphorbia jolkinii is mediated through the regulation of nitrogen transformation by functional microbial abundance in rhizosphere soils
Source: Front Microbiol. 2026 Feb 24;17:1757844. doi: 10.3389/fmicb.2026.1757844 (PMC12974137; doi:10.3389/fmicb.2026.1757844)
Supplement: Supplementary file 2 [file Table_2.DOCX]

**Supplementary methods**

1. **Detailed information on Kjeldahl method**

Soil total N content was determined using the Kjeldahl method. 1.0 g soil sample was weighed and placed into a digestion tube, along with one catalyst tablet and 10.0 mL of concentrated sulfuric acid. The mixture was thoroughly shaken, and 3 replicates were prepared for each treatment, with blank controls containing no soil. The digestion tubes were then placed in a graphite furnace at 420℃ for 60 min to digest. After digestion, the tubes were removed and cooled to room temperature. Analysis was conducted using a K1100 fully automatic Kjeldahl nitrogen analyzer (Kasite Experimental Instrument Co., Ltd., Shanghai) with the following settings: 25 mL of boric acid, 30 mL of dilution water, 40 mL of alkali solution, 5 min distillation time, titrant concentration of 0.02 mol/L. The total N content in the sample was calculated as the measured value minus the blank value.

1. **Detailed information on Chloroform fumigation method**

Soil microbial nitrogen (MBN) was determined following the method of Wang et al. (2024), with appropriate modifications. 100 g of fresh soil was placed in a beaker and adjusted with water to achieve a moisture content of 40% of field capacity. Each treatment sample was divided into steamed and unsteamed samples. Steamed samples were mixed with chloroform and 1 mol/L NaOH in a vacuum-sealed container, while unsteamed samples were placed in a sealed container containing 1 mol/L NaOH and incubated for 15 days. After incubation, nitrogen was extracted using K_2_SO_4_, digested, and analyzed for N content using a Kjeldahl nitrogen analyzer to calculate MBN.

**References**

Wang, Y.Z., Zhang, Y.P., Yang, Z.Y., Fei, J.C., Zhou, X., Rong, X.M., Peng, J.W., Luo, G.W., 2024. Intercropping improves maize yield and nitrogen uptake by regulating nitrogen transformation and functional microbial abundance in rhizosphere soil. Journal of Environmental Management 358, 120886.

Zhu, S.X.; Sun, S.X. Zhao, W., Sheng, L.Y., Mao, H., Yang, X.Q., Chen, Z.B., 2024. Metagenomics and metabolomics analysis revealed that Se-mediated Cd precipitation and nutrient cycling regulated soil-rice (Oryza sativa L) microenvironmental homeostasis under cadmium stress. Environmental and Experimental Botany 228, 105958.
